# Massive Gastrointestinal Bleeding Related to NSAID Use in a Patient with Ileorectal Anastomosis

**DOI:** 10.1155/2024/4619458

**Published:** 2024-08-30

**Authors:** Esere Nesiama, Letisha Mirembe, Kierra Weber, Sruthy Isaac, Deborah Trammell, Izi Obokhare

**Affiliations:** ^1^ University of South Carolina School of Medicine/Prisma Health Department of Orthopaedic Surgery, Columbia, USA; ^2^ Texas Tech University Health Sciences Center Amarillo Department of Surgery, Amarillo, USA; ^3^ University of Florida College of Pharmacy, Jacksonville, USA; ^4^ Texas Tech University Health Sciences Center School of Medicine, Lubbock, USA

## Abstract

Nonsteroidal anti-inflammatory drugs (NSAIDs) are commonly used to reduce pain and inflammation in over 30 million individuals daily. Gastrointestinal bleeding (GIB) associated with NSAID consumption has been well documented in gastric and duodenal bleeding; however, NSAID-associated GIB distal to the duodenum lacks extensive documentation. This report highlights small bowel occult bleeding related to NSAID use in a patient with a surgical history of robotic total colectomy with ileorectal anastomosis completed 1 year prior. In the case of bright red blood per rectum with associated NSAID use, we recommend NSAID cessation followed by an individualized treatment plan, such as upper/lower endoscopy and/or angioembolization.

## 1. Introduction

Nonsteroidal anti-inflammatory drugs (NSAIDs) are commonly used to relieve pain, inflammation, and high temperatures in over 30 million individuals each day across the globe [1]. Complications and treatment plans must be well documented to provide adequate care for cases that arise due to the usage rate. NSAID-induced enteropathy is the most common cause of occult GI bleeding over 40 years of age and is one of the most common complications of NSAID use [2]. In this report, we discuss the presentation and treatment of a patient who presented with bright red bleeding per rectum approximately 1-year postrobotic total colectomy with ileorectal anastomosis and concurrent NSAID use.

Enteritis is an inflammatory condition that can predispose the gastrointestinal (GI) tract to further inflammatory sequelae and damage. There are many different types of enteritis including eosinophilic, NSAID-induced, and infectious causes. Enteritis is associated with increased abdominal discomfort, endothelial dysfunction, mucosal barrier function, hypersecretion, and among other things [3]. Enteritis typically begins with localized epithelial inflammation but often progresses to more widespread inflammatory cascades. The inflammatory cascade of the innate immune system has been well documented in GI diseases as outlined by the *International Journal of Molecular Sciences* [4].

NSAID-induced enteritis is a specific form of enteritis attributed to the use of NSAIDs such as naproxen and motrin. Nonselective NSAIDs mechanism of action is via inhibition of the cyclooxygenase (COX) enzyme [5]. The COX enzyme is responsible for converting arachidonic acid into other eicosanoids, including prostaglandins [6], eliciting a therapeutic effect [5]. Prostaglandins play a crucial role in appropriate GI mucus production and blood flow [7]. Both COX-1 and COX-2 cyclooxygenase isoenzymes have an instrumental role in maintaining GI mucosal integrity and appropriate platelet and kidney function [5]. COX-1 is primarily involved in mediating gastric epithelium composition, while COX-2 plays a role in the inflammatory processes [8]. Nonselective NSAIDS cause direct irritation to the mucosa and inhibit protective prostaglandins [9]. Selecting a COX-2 selective NSAID will aid with inflammation while sparing the gastric mucosa [5, 7, 8].

Inflammation normally serves to protect the body from infection through eliminating microbes and enhancing the tissue healing processes. However, when prostaglandin synthesis is low as in NSAID-induced enteritis, the inflammatory response can be damaging [7, 9]. The mechanisms of NSAID-induced enteritis are thought to be due to several contributing factors. The phospholipid membrane of the mucosa is damaged directly by NSAIDs which consequently causes damage to the mitochondria. Mitochondrial damage results in a decrease in energy production, resulting in free radical generation. Damage to the intercellular junctions and increased permeability of the mucosa occurs. Intraluminal contents can then invade through the compromised mucosa and inflammation occurs [10]. Inflammation can compromise the intestinal mucosal epithelium, allowing for increased bacterial translocation and absorption of proteolytic enzymes, toxins, and bile acid [9]. Intestinal barrier damage leads to deleterious responses both locally and systemically. The mucosal epithelium can repair rapidly once aggravating factors are eliminated, as in our case; however, healing can be halted by the presence of nonselective NSAIDs [7].

## 2. Case Presentation

A 52-year-old female presented to our emergency room with complaints of “twenty-five bloody stools” over the course of ~24 hr. Her medical history was remarkable for chronic back pain for which she was taking self-prescribed Naproxen 500 mg twice a day by mouth for the past 3 months and chronic uncomplicated gastroesophageal reflux disease for which she was on Protonix 40 mg by mouth daily. Complete medication history at the time of presentation is recorded in Table 1.

The patient's social history included alcohol use: wine 1–2 drinks per week; tobacco: past use of cigarettes 20 per day, 13 years, last use 1998; and recreation drugs: denied. Allergies included a morphine with a visual hallucination reaction. No previous pregnancies and a history of hypertension in the patient's paternal side were noted. The patient's surgical history included a robotic total colectomy with ileorectal anastomosis performed 1 year prior to current admission for colonic inertia. Since her surgical colectomy, the patient reported a history of chronic diarrhea with over 10 bowel movements per day, with a recent onset of blood per rectum over the past several days. No other pertinent comorbidities or history was provided at the time of presentation.

Upon presentation, the patient endorsed nausea, vomiting, and mild bloating (ICD R10.9, K92.1). She denied any previous episodes of hematochezia, any recent travel, or sick contacts. Fecal occult blood test was positive. Computerized tomography (CT) results were suggestive of an active bleed within the left colon ~26–30 cm proximal to the anus. The patient was admitted on a Friday night for observation and serial labs. Inpatient medications are listed in Table 2.

Gastroenterology was consulted for possible colonoscopy and interventional radiology for possible embolization, and intervention was planned for the following week if she remained hemodynamically stable over the weekend. Her admission hemoglobin of 12.7 gm/dL and hematocrit of 39% making this case nonemergent. Serial hemoglobin and hematocrit were ordered and stable within normal limits throughout the weekend.

**Figure 1:**
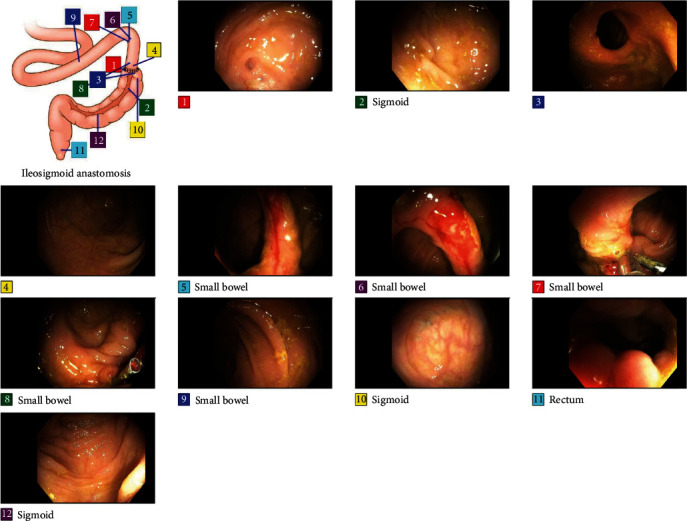
Endoscopy images taken during the lower endoscopy 2 days after admission.

**Figure 2:**
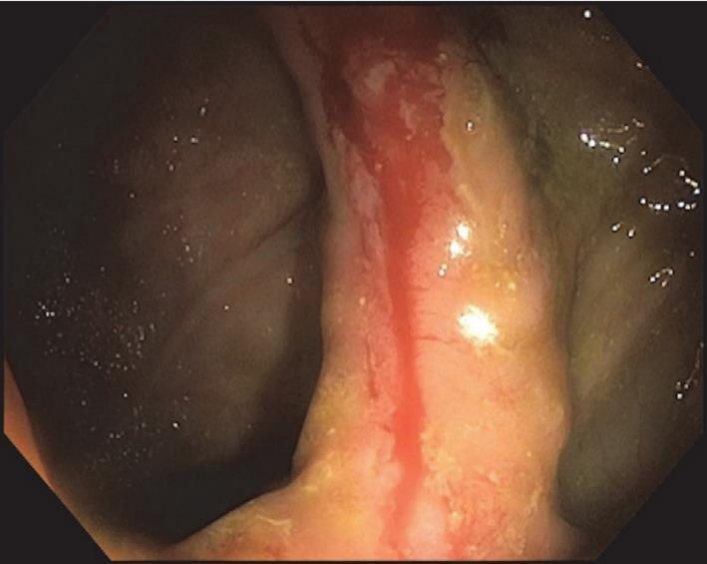
Endoscopy image at the level of the ileorectal anastomosis revealing friable, bleeding epithelial surface. This image is a close-up image corresponding to number 5 in Figure 1.

**Figure 3:**
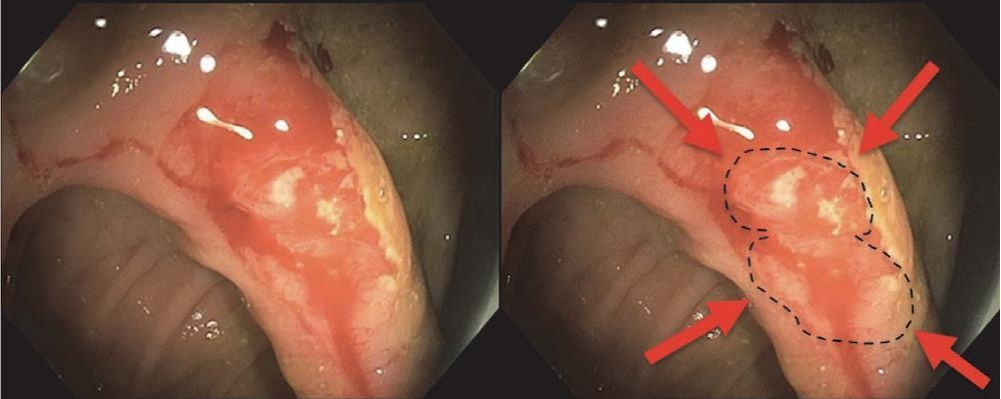
Endoscopy image at the level of the ileorectal anastomosis revealing a pill-shaped indentation at the center of the punctate bleeding. Side-by-side image provides an outline of the pill-shaped indentations with arrows for clarity. This image is a close-up image corresponding to number 6 in Figure 1.

**Figure 4:**
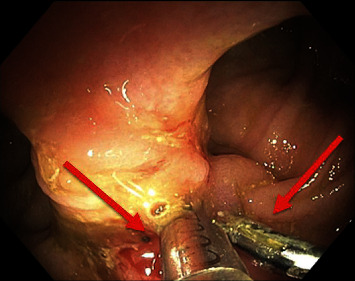
Endoscopy image at the level of the ileorectal anastomosis with arrows pointing to the placement of hemostatic clips used to stop the bleed. This image is a close-up image corresponding to number 7 in Figure 1.

Colonoscopy was performed 2 days after admission (Figure 1). The colonoscope (Olympus PCF-H180AL) was advanced from the anus to the mid-ileum beyond the anastomosis. The anastomosis appeared patent. The quality of the bowel preparation was adequate to identify polyps 6 mm in size and larger. Adjacent and proximal to the level of the anastomosis was an area of active bleeding and friable tissue (Figure 2). Close-up examination of the anastomosis revealed multiple pill-shaped indentations on the epithelial surface at the center of the bleed (Figure 3). The area was clipped using seven endoscopic hemostatic clips as shown in Figure 4. Bleeding was controlled and the patient tolerated the procedure well (ICD 45382). The rest of the colonoscopy revealed normal healthy tissue on direct and retroflexion views (Figure 1). Serial labs including hemoglobin and hematocrit remained stable during the hospital stay. The patient was discharged on hospital day 5. Two-week follow-up in clinic was advised, and lower endoscopy was planned in 3–6 months to ensure complete healing. The results of postoperative follow-up revealed complete resolution of GI bleeding at the first follow-up and return to normal bowel movements by the 6-month return visit. The patient was encouraged to abstain from NSAIDs and given iron supplementation.

## 3. Discussion

Our case demonstrates a hemodynamically stable patient with a GIB at the level of a previous ileorectal anastomosis due to an invagination of NSAID from chronic NSAID use. Naproxen maximum dosage as defined by regulatory institutions such as the World Health Organization and other entities is usually limited to 500 mg two to three times daily with a maximum total daily dose of 1,500 mg if prescribed by a healthcare provider but not to exceed 660 mg if self-prescribed as in our case [9]. Due to the lack of drug-metabolizing genetic studies, the effect of possible CYP2C9 alleles was not evaluated [10]. Due to the stability of the patient, they were managed nonurgently with a pediatric endoscope which revealed the punctate bleeding with a pill-shaped spot in the epithelium at the level of the anastomosis. The bleed was managed by hemostatic clips, and preemptive cessation of NSAIDs was recommended to prevent recurrent bleeds. There was complete resolution with no hematochezia reported at postoperative follow-ups 1-year status post.

High worldwide usage rates of NSAIDs warrant the early suspicion and evaluation of patient presenting with GIB. GIB associated with NSAID consumption is well documented for stomach and duodenal injuries with treatment plans focused on imaging, cessation of the offending agent, and the administration of proton pump inhibitors (PPIs). However, there is minimal amount of literature describing the presentation and treatment of midgut GI bleeding. Based on recent endoscopic studies, the estimated prevalence associated with different types of GIB among NSAID users are presented in Table 3 [11].

The prevalence of lower GI enteropathy due to chronic NSAID use in recent years warrants prompt exploration to provide targeted management in cases with occult and overt GI bleeding and iron deficiency anemia, as seen in this case. Epidemiological trend lines suggest that there has been a decreased prevalence of upper GI complications while prevalence of lower GI complications associated with chronic NSAID use has increased, further emphasizing the need for a universal management protocol [12].

The pathogenesis of NSAID-associated enteritis is unclear; however, it is assumed to be multifactorial as detailed by the three-hit hypothesis proposed by Bjarnason et al. [12] and typically presents as abdominal pain, diarrhea, protein loss, and iron deficiency anemia when associated with bright red bleeding per rectum [13]. Since these findings are nonspecific, further imaging and testing are needed for diagnosis and management. Depending on patient presentation and hemodynamic stability, specific steps should be taken for management that are in line with current protocols for unknown gastrointestinal bleeding (GIB). According to the recent American College of Gastroenterology (ACG) guidelines published in February 2023, patients with lower gastrointestinal bleeding (LGIB) who are hemodynamically stable can undergo colonoscopy with bowel preparation for lower GI tract analysis. In patients who are moderately stable, a colonoscopy with subsequent esophagogastroduodenoscopy (EGD) is sufficient for initial management [14]. EGD is done to exclude brisk upper gastrointestinal bleed as the cause of hemodynamic instability [15]. Patients who are hemodynamically unstable despite medical interventions should be resuscitated followed by colonoscopy without bowel preparation and upper endoscopy to determine the source of bleeding [14]. ACG guidelines recommend computed tomography angiography (CTA) in hemodynamically unstable patients with ongoing hematochezia, but it is not recommended in those with minor LGIB. If CTA shows extravasation, then it can be promptly treated with angioembolization [14, 16, 17, 18].

**Figure 5:**
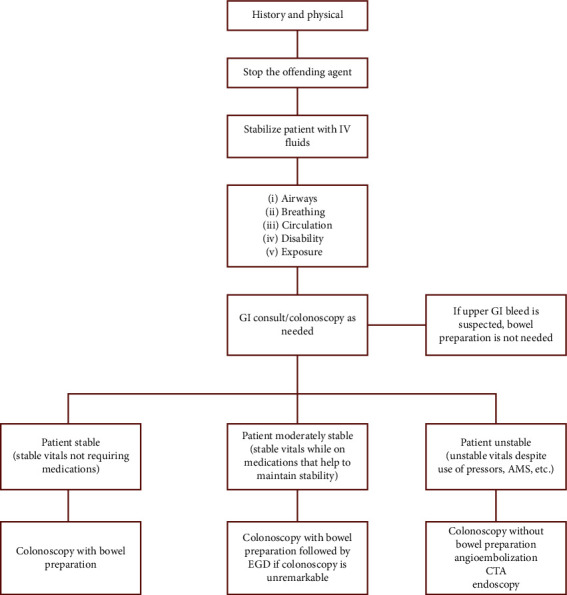
The flowchart proposed standard of care for occult GI bleeding in a patient with an ileorectal anastomosis.

Since there is no universally agreed upon method for prevention or management of this patient's specific presentation, we endorse NSAID cessation as a primary treatment option. There are no convincing studies on the rest of the patient's medication list that would contribute to their presentation. Due to the lack of efficacy of current drug regimens under investigation for lower GI and gastric injuries (PPIs, selective COX-2 inhibitors, and sulfasalazine), further pharmacological and clinical studies are needed to identify effective treatment and prevention methods specific to cases involving lower GI bleeds [13]. Although there is promising data surrounding the use of Rebamipide and Eupatalin for the treatment of intestinal inflammation, more data collection is necessary before recommendations can be safely implemented [13]. Our proposed standard of care is outlined in Figure 5.

Hemodynamically stable patients with rectal bleeding can have a colonoscopy along with bowel preparation for lower GI tract analysis (Figure 5). Managing the underlying cause would be the first step if LGIB is diagnosed on colonoscopy and the cause of the bleeding is identified. In our case, the bleeding was secondary to NSAID use and therefore the initial step was to stop the offending drug. In about 75%–85% cases of LGIB, the symptoms will resolve with supportive care only, but additional measures should be taken to stop or minimize the bleeding if the symptoms persist or recur [19]. The primary purpose of colonoscopy is to identify the bleeding site; however, it can be also utilized for therapeutic management. Contact cautery, argon plasma, or laser coagulation are a few modalities documented to be effective during colonoscopy to stop LGIB [16, 19].

Based on the patient's hemodynamic stability, the source of the bleeding, and the degree of intestinal mucosal injury, elective or emergency procedures can be performed to manage the LGIB. Elective procedures include endoscopic or endovascular techniques. Surgical resection is considered emergent intervention and is generally reserved for hemodynamically unstable patients in whom other bleeding cessation methods have failed and/or the bleeding site is unknown [19]. Less invasive procedures include endovascular management and angioembolization.

Endovascular management of bleeding can be achieved through angiographic embolization or vasoconstriction via vasopressin infusion [18, 19]. Vasopressin infusion causes decreased blood flow to the area of the bleed but there is an increased risk of rebleeding once the vasopressin infusion is stopped [19]. Angiographic embolization is another widely used treatment modality for LGIB but there is an increased risk of colonic infarction associated with this technique [18]. To reduce this risk, transcatheter superselective embolization technique is used in which microcatheters target the subsegmental peripheral arterial branches for embolization [19]. Compared to other vascular intervention techniques, the transcatheter superselective embolization method is considered to have a better safety profile, lesser adverse effects like mucosal ischemia, lower recurrence rate, and is more efficacious [19]. Hence, superselective embolization is now considered first-line angiographic therapy for lower GI bleeding. Since there are many factors to account for, we endorse the use of our standard of care guidelines for occult GI bleeding in patients with an ileorectal anastomosis (Figure 5). Although our case is very rare, it emphasizes the importance of medication counseling to patients after anastomosis cases. Repetitive education and monitoring at follow-up visits with postoperative anastomosis patients and colorectal surgery patients in general can mitigate adverse effects from gastroerosive medication abuse, preventing events like this from occurring in the future.

## Figures and Tables

**Table 1:** Home medication list of the patient upon presentation to our medical facility.

Home medication	Dosage
Ascorbic acid (Vitamin C 1,000 mg oral tablet)	1,000 mg one tab by mouth two times a day
Cetirizine (cetirizine 5 mg oral tablet)	5 mg one tab by mouth daily
Estradiol topical (estradiol 0.1 mg/g vaginal cream)	1 g vaginal at bedtime three times a week
Fluorometholone ophthalmic (Flarex 0.1% ophthalmic suspension)	One drops both ears as directed
Fluticasone nasal 0.05 mg (Flonase)	Two sprays nasal inhalation daily
Gabapentin (gabapentin 300 mg oral capsule)	300 mg one capsule by mouth three times a day
Lifltegrast ophthalmic (Xiidra 5% ophthalmic solution)	One drop both eyes two times a day
Magnesium oxide (magnesium oxide 400 mg (240 mg elemental magnesium) oral tablet)	One tab by mouth two times a day
Methocarbamol (methocarbamol 750 mg oral tablet)	750 mg one tab by mouth every 8 hr as needed
Potassium drops	Two drops by mouth daily
Iron patch	Two patches transdermal daily
Prenatal gummies	One gummy by mouth two times a day
Omega-3 polyunsaturated fatty acids (fish oil oral capsule)	One capsule by mouth daily
Pantoprazole (pantoprazole 40 mg oral delayed-release tablet)	40 mg one tabs by mouth daily
TiZANidine (tiZANidine 4 mg oral tablet)	4 mg one tab by mouth at bedtime
TraMADol (traMADol 50 mg oral tablet)	100 mg two tabs by mouth three times a day as needed
Zolpidem (Ambien 10 mg oral tablet)	10 mg one tab by mouth at bedtime

**Table 2:** Inpatient medication list of the patient upon presentation to our medical facility.

Inpatient medications	Dosage
Cetirizine 5 mg tab (cetirizine)	5 mg one tab, oral, daily
Fluticasone nasal 0.05 mg (flonase)	0.1 mg two sprays, nasal, daily
Gabapentin 300 mg cap (gabapentin)	300 mg one tab, oral, TID
Pantoprazole 40 mg oral EC tab (pantoprazole)	40 mg one tab, oral, daily
TiZANidine 4 mg tab (tiZANidine)	4 mg one tab, oral, qHS
Continuous lactated ringers 1,000 mL	1,000 mL, IV, 125 mL/hr
PRN methocarbamol 750 mg tab (methocarbamol)	750 mg one tab, oral, qSH
PRN traMADol 50 mg tab (traMADol)	100 mg two tabs, oral, TID

**Table 3:** Prevalence of NSAIDs associated with GI injuries based on endoscopic studies [10].

Complication	Prevalence in chronic NSAID users (%)
Small intestine enteropathy	71
Gastric ulcers	15
Duodenal ulcers	10
Small intestine ulcers	8.4
Bleeding or perforation	2–4

## Data Availability

The data that support the findings of this study are available from the corresponding author upon reasonable request.
